# Current treatment of multidrug resistant tuberculosis in Ethiopia: an aggregated and individual patients’ data analysis for outcome and effectiveness of the current regimens

**DOI:** 10.1186/s12879-018-3401-5

**Published:** 2018-09-27

**Authors:** Setegn Eshetie, Animut Alebel, Fasil Wagnew, Demeke Geremew, Alebachew Fasil, Ulrich Sack

**Affiliations:** 1 Department of Medical Microbiology, School of Biomedical and Laboratory Sciences, College of Medicine and Health Sciences, University of Gondar, Gondar, Ethiopia; 2 Department of Clinical Nursing, College of Health Sciences, Debre Markos University, Debre Marqos, Ethiopia; 3 Department of Immunology and Molecular Biology, College of Medicine and Health Sciences, University of Gondar, Gondar, Ethiopia; 4 Department of Clinical Chemistry, College of Medicine and Health Sciences, University of Gondar, Gondar, Ethiopia; 5 Institute of Clinical Immunology, Medical Faculty, University of Leipzig, Leipzig, Germany

**Keywords:** Ethiopia, MDR-TB, Treatment success

## Abstract

**Background:**

The programmatic management of Multidrug-resistant tuberculosis (MDR-TB) is entirely based on a WHO recommended long-term, 18–24 month lasting treatment regimen. However, growing evidence shows that low treatment success rate and high rates of adverse events are associated with this regimen. Up to date, the MDR-TB treatment outcome is not sufficiently understood in Ethiopia. Therefore, this analysis aimed to determine the pooled estimates of successful (cure, completed, or both), and poor outcomes (death, failure, and lost to follow ups).

**Method:**

A systematic search was performed to identify eligible studies reporting MDR-TB treatment outcomes in Ethiopia. Relevant studies for our analysis were retrieved from PubMed database search, Google Scholar and institutional repository sites of Ethiopian universities up to March 15, 2018. The primary outcome was treatment success, referring to a composite of cure and treatment completion. A random effect model was used to calculate pooled estimates.

**Results:**

Six studies reporting treatment outcome on the 1993 MDR-TB patients were included in this analysis. Of the cases, the 1288 and 442 patients had a successful and poor outcome, respectively. In the pooled analysis, treatment success was observed in 59.2% (95%CI, 48.1–70.4) of patients, while 23.3% (95%CI, 19.7–27.0%) of patients had a poor outcome. in sub-group analysis,46.1% (95%CI, 34.2–58.0) were cured, 12.8% (5.7–20.0) treatment completed, 14.3% (11.5–17.2) died, 7.5% (3.7–11.3) lost to follow up, and 1.6% (1.1–2.2%) experienced treatment failure. The 25.0% (14.6–35.5) patients whose treatment outcome was not assessed (on treatment or transfer-out).

**Conclusion:**

The result of this study highlight treatment success among MDR-TB is below acceptable range. To update the current treatment regimen, the levels of evidence need to be replicated through meticulous surveillance systems.

**Trial registration:**

Study protocol registration: CRD42018090711.

**Electronic supplementary material:**

The online version of this article (10.1186/s12879-018-3401-5) contains supplementary material, which is available to authorized users.

## Background

Multidrug-resistant tuberculosis (MDR-TB) is defined by resistance to at least the two most powerful first line anti-tuberculosis drugs, isoniazid, and rifampicin [1]. MDR-TB remains a major public health crisis, with an estimated 490, 0 000 new cases are emerging globally each year [2]. The increasing incidence of drug resistant TB is mainly reported from resource-limited countries where TB care programs are compromised. Globally, 3.9% (95% CI 2.7–5.1%) of new cases and 21% (95% CI 15–28%) of previously treated TB cases were in MDR-TB cases. It is also estimated that each year 580,000 and 250,000 deaths reported among new and previously treated MDR-TB cases [3], respectively. According to the recent global report, 2900 (1800–4000) MDR-TB cases were estimated among notified pulmonary TB in Ethiopia [2]. A latest meta-analysis study also reported that 2% of new cases and 17% among previously treated cases were MDR-TB patients [4].

Programmatic management of drug-resistant TB treatment is based on conventional or longer MDR/ rifampicin resistant (RR)-TB treatment regimens, which lasts for 20–24 months and achieved a success rate nearly in 50% of patients, worldwide [5]. The low successful treatment outcome might be due to prolonged treatment period, low drug efficacy and toxic regiments. Recent advances show that high treatment success was achieved by using a short course treatment regimen. To date, few studies have been conducted to reveal the effectiveness of short-course MDR/or RR-TB treatment regimen. The recent meta-analysis studies showed that the treatment success rate using a shorter MDR/RR-TB treatment regimen was ranged from 83.0–83.7% [6–8], which is significantly higher than a meta-analysis reported a success rate for conventional, longer MDR/RR-TB treatment regimen (54%) [9]. Moreover, the effectiveness of shorter course MDR/RR-TB treatment was also explored in other institutional and observational studies. A study from nine African countries revealed that the treatment success and cure rate of short MDR/RR-TB regimen was 81.6% and 72.4%, respectively [10]. Similarly, promising reports for short course MDR/RR-TB treatment were also noted in Bangladesh, Niger, and Cameron [11–14].

Generally, the existing facts asserted that the safety and effectiveness of the shorter MDR/RR-TB regimen are superior compared to the previously appreciated reports for the longer MDR/RR-TB regimen. Even more, a 9–12 month regimen for MDR/RR-TB was also recommended by World Health Organization (WHO) [15]. But, prior to putting this regimen in place or before this regimen can be prescribed for the MDR/RR-TB patients in a given country, the level evidence in both regimens needs to be replicated and supported through rigorous research and with a large number of patients, in order to conclude that this most effective treatment for MDR/RR-TB. Besides, still findings are based on observational design, and therefore future researches need to be focus on high-level designs such as randomized controlled trials (RCTs) to generate highly relevant evidence in this regard. Most importantly, this short course treatment should not completely replace the conventional treatment approach since the fact that patients with fluoroquinolones or other 2nd line drug resistance, extra-pulmonary tuberculosis, pregnancy and with severe clinical problems are known not to be considered for shorter MDR/RR-TB regimen. In this case, the national TB control program (NTPs) in need to maintain the conventional treatment approaches.

The MDR-TB treatment in Ethiopia is mainly based on a standard or long course treatment, which is known as less effective to achieve high levels of successful outcome. The Ethiopian Federal Ministry of Health adopted a standardized regimen consists of an 8-month intensive phase with a combination of pyrazinamide, capreomycin, levofloxacin and prothionamide or ethionamide and cycloserine, a 12-month continuation phase with a combination of pyrazinamide, levofloxacin,prothionamide or ethionamide and cycloserine [16].

By far, few observational studies have been conducted to reveal treatment outcomes among MDR-TB patients, and even the findings are inconsistent and representing only local information. Hence, the effectiveness of the standard MDR-TB treatment was not fully assessed in Ethiopia. Therefore, we report here a meta-analysis of observational studies of regimens of 18–24 months in duration whose composition was based on a standardized longer term regimen. The purpose of the study was first, to determine the pooled successful and unfavorable treatment outcome measures. Secondly, the sub-group analysis was also done to estimate the proportion of cured and treatment completed cases among successfully treated cases, and to evaluate poor treatment outcomes including treatment failures, deaths, and lost to follow ups.

## Methods

### Studies/setting

Studies that reported the treatment outcome among MDR-TB patients in Ethiopia were included for this analysis. However, studies focused on non-MDR or drug-susceptible TB patients were not eligible for this study.

### Study protocol registration

The study has been registered in PROSPERO database with protocol number, CRD42018090711.

### Intervention

The original studies reported an outcome from patients treated with a longer, 18–24 months MDR-TB treatment regimen.

### Comparison

MDR-TB treatment in Ethiopia is largely based on a standardized longer regimen. Therefore, no comparison was undertaken for this analysis.

### Outcomes

MDR-TB treatment outcomes were evaluated as treatment success and unfavorable outcome. Successful outcomes described as patients meeting the definition of Cure or treatment completed. Poor treatment outcomes refer to patients meeting the definition of death, lost to follow up, and treatment failed.

### Search strategy and quality evaluation

The PubMed database was employed to retrieve the available research reports in Ethiopia. A highly sensitive search strategy was developed using the combination of the following Keywords; treatment outcome AND multidrug-resistant tuberculosis OR drug-resistant tuberculosis OR MDR-TB AND Ethiopia, both as exploded as MESH headings and free-text terms. Manual searching was also done from the institutional repository websites of Ethiopian universities (such as Addis Ababa University, and University of Gondar) to include unpublished reports. Besides, grey literature searching was also performed using Google Scholar database to collect non-PubMed indexed articles. Electronic database searches were conducted in February and March 2018. Two authors (SE, AE) reviewed all abstracts and a full-text article, with the final decision, was made through consensus. In cases of disagreement, the consensus was achieved through arbitration of other authors.

Studies were potentially acceptable if they report the treatment outcome on at least 100 MDR-TB patients within a defined study design approach, retrospective, and prospective cohorts. The original studies were also rated as good quality when the average treatment duration was 12 months and above. However, treatment outcome other than MDR-TB was not examined in our meta-analysis. The overall quality score of the included studies was measured using the Newcastle-Ottawa quality assessment scale for observational studies (Additional file 2: Table S2).

### Data extraction and statistical analysis

Two investigators (SE, AA) extracted data using a standard abstraction form. In the case of disagreement, other investigators (FW, US) took part to resolve the differences through discussion. Data abstraction was carried out with regard treatment outcomes. The primary outcome of this study was treatment success included cure, treatment completed, or both. The secondary outcome of the study was poor outcomes (such as, lost to follow up, death and failure). Moreover, for each included study the following information was also collected; HIV status, and previous history of anti-TB treatment.

The pooled proportions of the treatment outcomes with 95%CI were statistically measured using Stata version 11.0 software (College Station, TX: StataCorp LP). A Freeman-Tukey-type arcsine square-root transformation and DerSimonian and Laird random-effects model were used to stabilize variances and calculate pooled estimates [17].The sensitivity analysis on primary outcome was done using a Bayesian random-effects model with Monte Carlo Markov chain simulations of variability [18]. The I^2^ statistic was used to assess the proportion of overall variation attributable to between-study heterogeneity [19].

## Results

### Selection process and description of the included studies

**Fig. 1:**
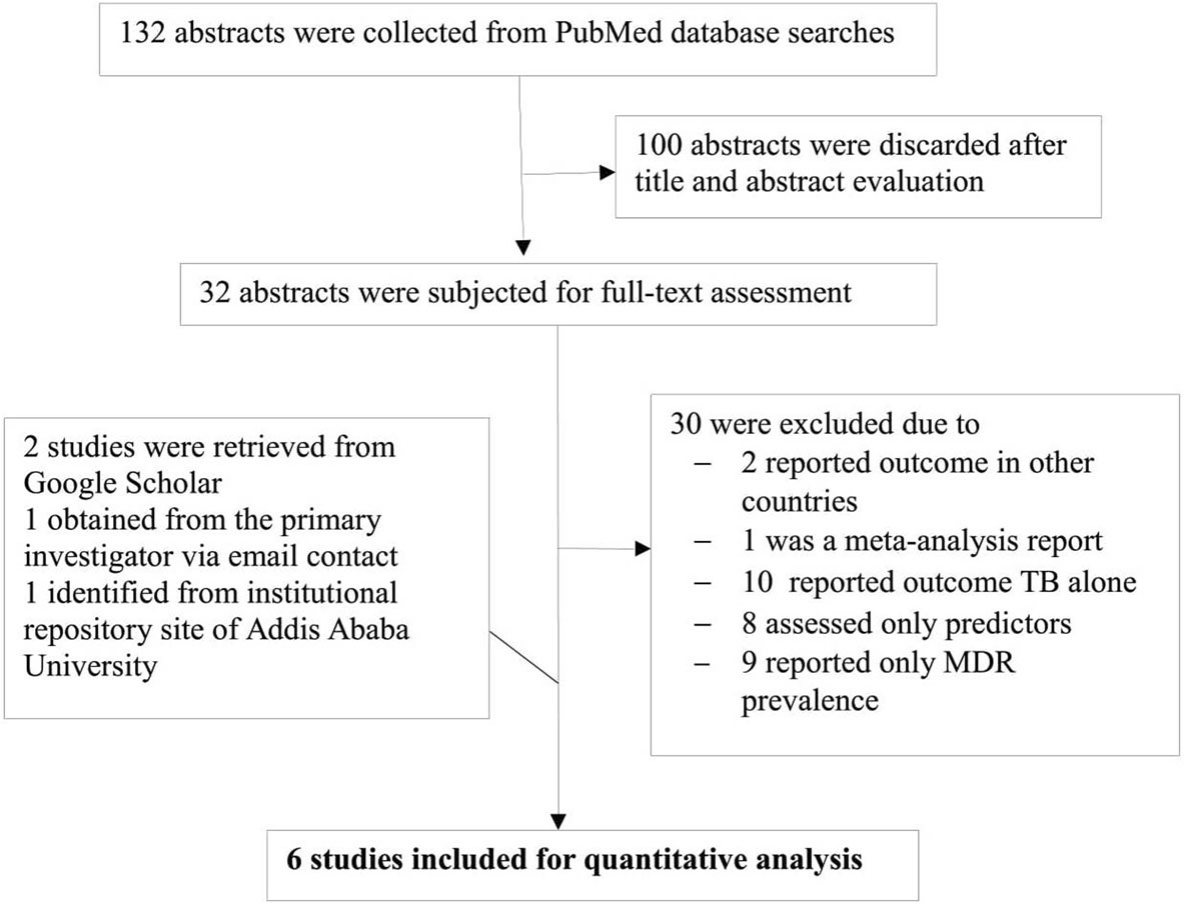
Study selection process

The selection process is depicted in Fig. 1, a total of 132 abstracts were identified from an initial electronic database search. Following title and abstract evaluation, 100 citations were excluded and the remaining 32 were subjected to full-text evaluation. After full-text review, 2 studies were found to be eligible for the analysis and a further 4 studies were obtained through manual searching, institutional repository sites of Addis Ababa University and email contact from the primary investigator. Finally, 6 studies were found to be acceptable for our analysis [20–25].

The baseline characteristics of the studies were indicated Table 1, of the included studies, three were retrospective cohorts, and the remaining were descriptive retrospective chart reviews and prospective cohort studies. All studies reported the patients’ previous history of anti-TB drug treatment and HIV co-infection. The included studies reported treatment outcome among 1993 MDR-TB patients representing Northwestern, Northeastern, Central, Eastern and Southern Ethiopia, MDR-TB care centers. Of the patients, successful treatment outcome was observed in 1288 patients, while 442 were poorly treated cases (Table 2). However, the treatment outcome of 263 patients was not evaluated; this was due to either the transfer out or patients on the course of treatment. Relatively high treatment success rate was reported in Meressa et al. (78.6%) [25] followed by Tolera et al. (65.9%) [24] and Alene et al. (63.6%) [22]. On the other hand, a high proportion of patients with poor outcome was observed in Tolera et al. (34.1%) [24], followed by Alene et al. (25.6%) [22] and Baye et al. (22.7%) [20].

**Table 1:** The baseline characteristics of the included studies

Study area	First author, year of publication	Study period	Study design	No of MDR-TB cases	Types of cases	Method	Treatment category	HIV status
Northeastern Ethiopia	Baye et al, 2018 [20]	2012–2016	RC	141	Pulmonary tuberculosis (96.5%) Extra-pulmonary tuberculosis (3.5%)	Line probe assay Culture Xpert MTB/RIF	New (8.5%) Relapse (13.5%) After lost to follow up (2.1%) Failure of a new regimen (30.5%) After the failure of retreatment (42.6%) Transfer in from other sites (1.4%) Other (1.4%)	HIV co-infected, 38 (27%), the majority of the patients have received ART (84.2%)
Southern Ethiopia	Girum et al*,* 2017 [21]	2009 to 2014	RC	154	Pulmonary tuberculosis (94%) Extra-pulmonary tuberculosis (6%)	Culture Xpert MTB/RIF	History of previous treatment (90%) New cases (10%)	HIV co-infection, 11 (7.2%), of them 9 have had a history of previous treatment
Northwest Ethiopia	Alene et al, 2017 [22]	2010 to 2015	RC	242	Pulmonary tuberculosis (94%) Extra-pulmonary tuberculosis (6%)	Culture Xpert MTB/RIF	History of previous treatment (93%) New cases (7%)	HIV co-infection, 51 (21.1%)
Central Ethiopia	Mequanint et al., 2014 [23]	2011 to 2013	DRC	680	Pulmonary tuberculosis (84%) Extra-pulmonary (15.6%) Both, 0.4%	Culture	History of previous treatment, 35%	HIV co-infection, 193 (28.4%)
Eastern Ethiopia	Tolera et al. 2018 [24]	2005 to 2016	DRC	164	Pulmonary tuberculosis (98.2%) Extra-pulmonary tuberculosis (1.8%)	–	Retreated cases, 97%	HIV co-infection, 41 (25%), the majority of the patients have received ART (97.6%)
Central and Northwest Ethiopia	Meressa et al., 2015 [25]	2009 to 2014	PC	612	Pulmonary tuberculosis (93%) Extra-pulmonary tuberculosis (7%)	Culture Line probe assay	History of previous treatment (98.5%) HIV infection (21.7%)	HIV co-infection, 133 (21.7%), the median CD4 count was 239 cells/ml^3^, the majority of the patients have received ART (90.2%)

Key: *RC* retrospective cohort study, *DRC* descriptive retrospective cross-sectional study, *PC* prospective cohort study

**Table 2:** MDR-TB treatment outcome extracted from the original studies

First author and year of publication	Study area	Successful outcome			Unfavorable outcome				Not evaluated		
		Cured, n (%)	Treatment completed, n (%)	Total, N (%)	Died, n (%)	Lost to follow up, n (%)	Treatment failed, n (%)	Total, N (%)	Transfer out, n (%)	On treatment, n (%)	Total, N (%)
Baye et al., 2018 [20]	Northeastern Ethiopia	55 (39.0)	3 (2.1)	58 (41.1)	22 (68.8)	8 (15.6)	2 (1.4)	32 (22.7)	–	–	51 (36.2)
Girum et al, 2017 [21]	Southern Ethiopia	39 (25.3)	26 (16.9)	65 (42.2)	13 (8.4)	20 (13.0)	0	33 (21.4)	5 (3.2)	50 (32.5%)	56 (36.4)
Alene et al, 2017 [22]	Northwest Ethiopia	131 (54.1)	23 (9.5)	154 (63.6)	31 (12.8)	27 (11.2)	4 (1.7)	62 (25.6)	6 (2.5)	20 (8.3)	26 (10.7)
Mequanint et al., 2014 [23]	Central Ethiopia	274 (40.3)	148 (21.8)	422 (62.1)	105 (15.4)	9 (1.3)	14 (2.1)	128 (18.8)	8 (1.2)	122 (17.9)	130 (19.1)
Tolera et al., 2018 [24]	Eastern Ethiopia	86 (52.4)	22 (13.4)	108 (65.9)	37 (22.6)	17 (10.4)	2 (1.2)	56 (34.1)	0	0	0
Meressa et al., 2015 [25]	Central and Northwest Ethiopia	396 (64.7)	85 (13.9)	481 (78.6)	85 (13.9)	36 (5.9)	10 (1.6)	131 (21.4)	0	0	0

### MDR-TB treatment outcomes

**Fig. 2:**
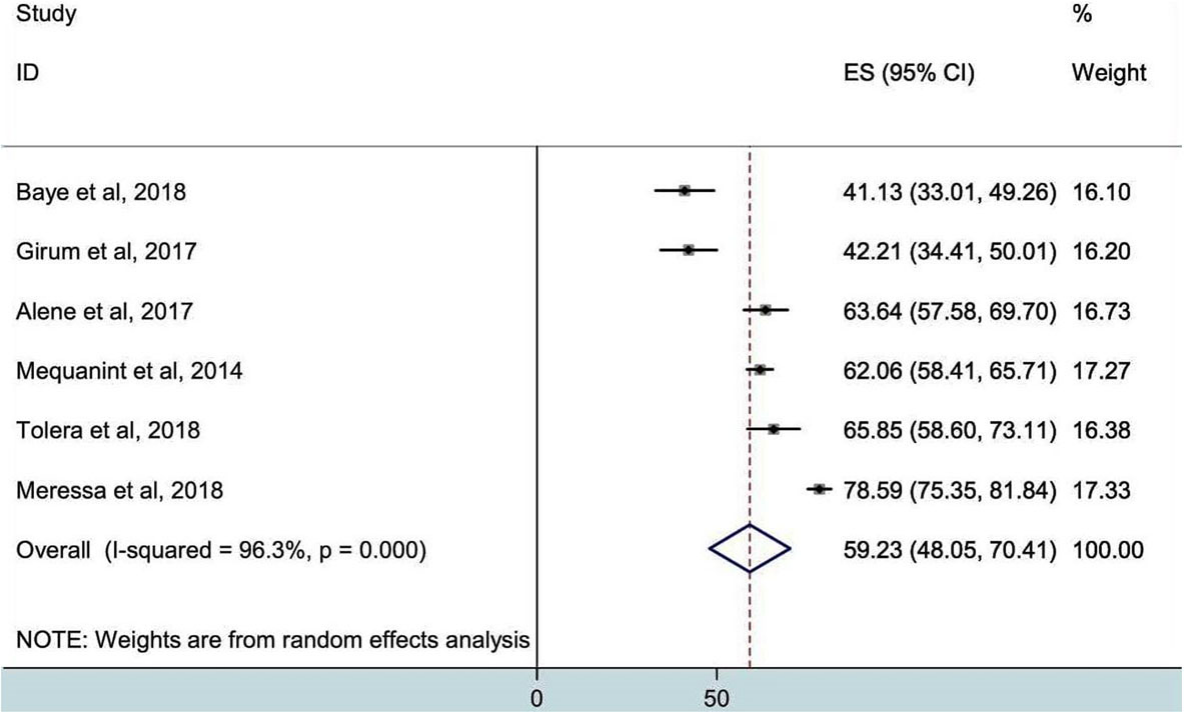
Pooled estimates of successful treatment outcome

**Fig. 3:**
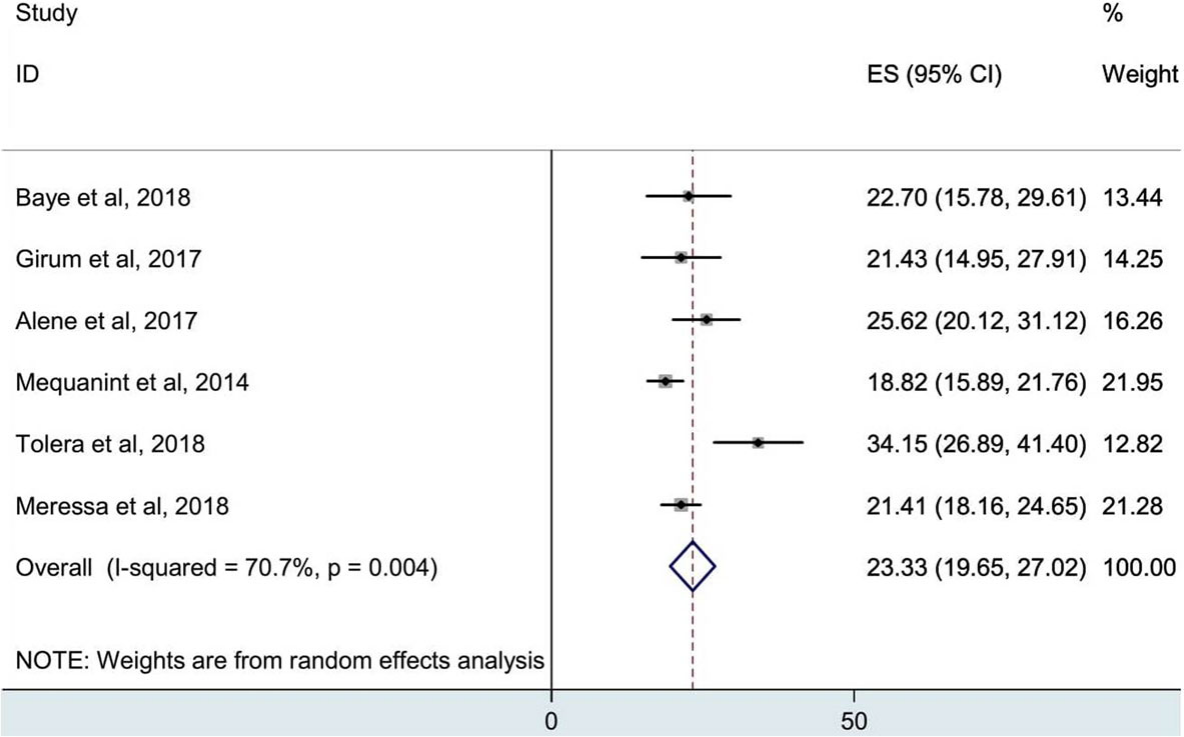
A pooled estimate of poor treatment outcome

In the present analysis, the pooled treatment success was estimated as 59.2% (95%CI, 48.1–70.4). The high heterogeneity between studies was observed (I^2^, 96.3%) (Fig. 2), but evaluation of the publication bias is not recommended for a meta-analysis of lower than 10 studies. Likewise, the overall poor treatment outcome was also determined, as seen in Fig. 3, 23.3% (95%CI, 19.7–27.0%) of the patients were unsuccessfully treated. High heterogeneity was also indicated (I^2^, 70.7%).

**Fig. 4:**
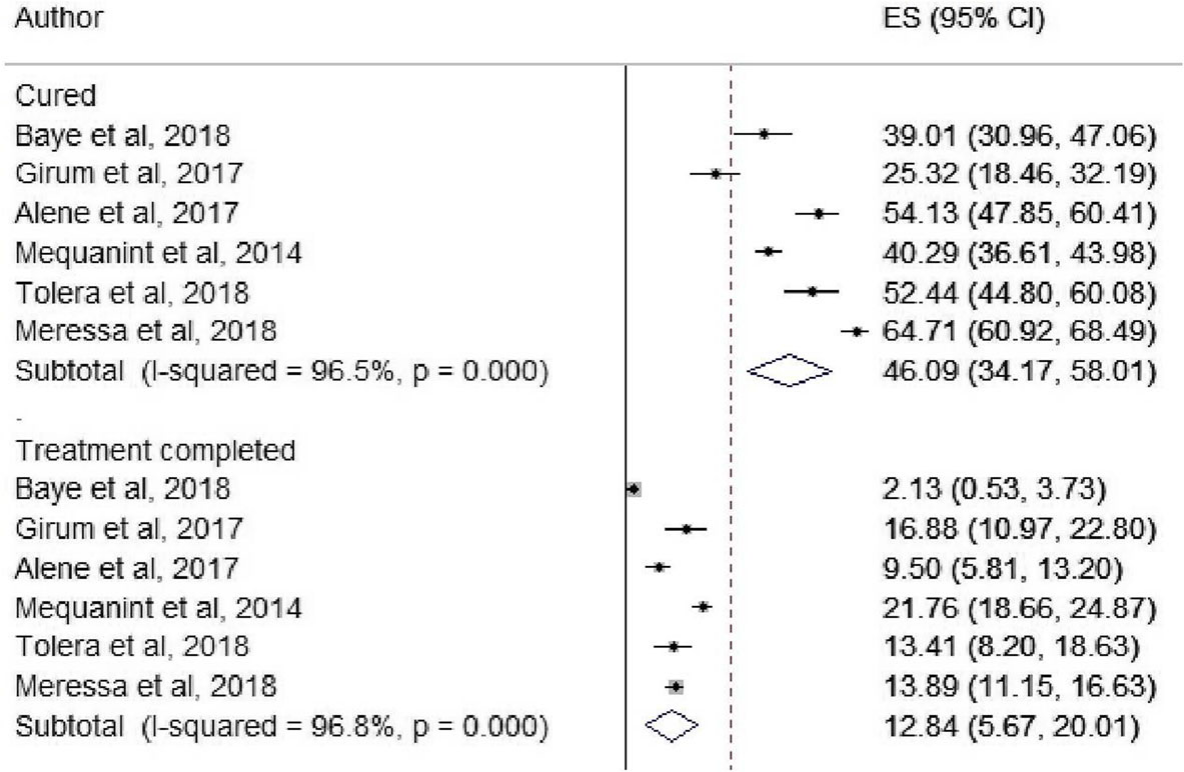
The pooled proportion of cured and treatment completed cases

**Fig. 5:**
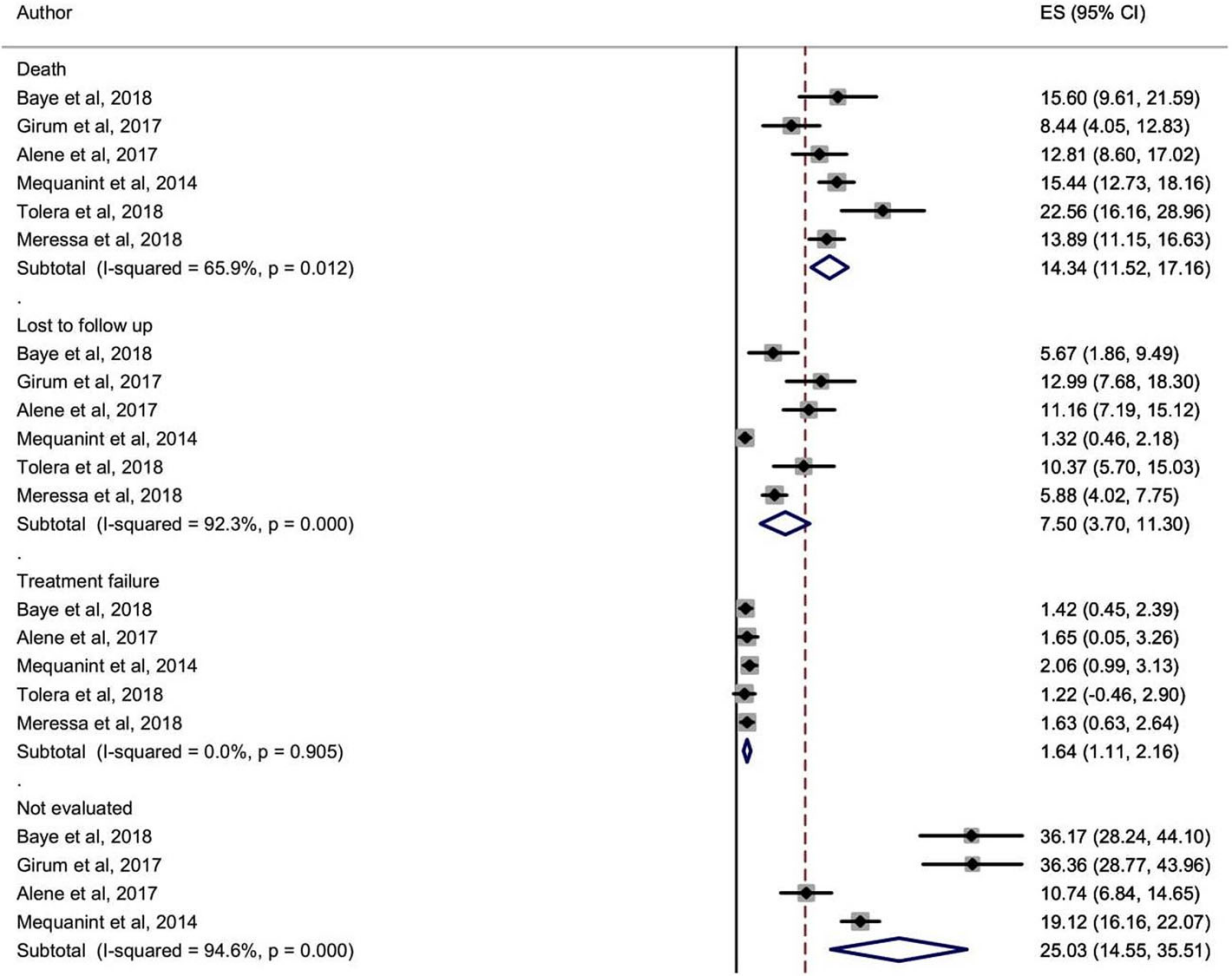
The pooled proportion of died, lost to follow up, patients experienced treatment failure, and patients whose outcome was not evaluated

In sub-group analysis, 46.1% (95%CI, 34.2–58.0) and 12.8% (95%CI, 5.7–20.0) of MDR-TB patients were cured and treatment completed, respectively (Fig. 4). Moreover, the pooled proportion of poor treatment outcomes was also presented in Fig. 5, 14.3% (95%CI, 11.5–17.2) died, 7.5%(95%Cl, 3.7–11.3) lost to follow up, and 1.6% (95%Cl, 1.1–2.2%) were patients with treatment failure. In Fig. 5, 25.0% (95%Cl, 14.6–35.5) of the MDR-TB patients whose treatment outcome was not examined, as noted in the original studies, these were patients on treatment or transfer-out.

## Discussion

Though the WHO task force set a strategic plan to target of 75% successful treatment outcome of MDR-TB by the end of 2015 in global bases [26], many countries including Ethiopia have not yet achieved this ambitious aim. Maximizing the favorable treatment outcome in MDR-TB is a global health priority and one of the key performance indicators of WHO’s End TB strategy [27]. Ethiopia has made the treatment of MDR-TB a national health priority [28].

The national MDR-TB treatment program based on the recommendations from the 2011 update of Guidelines for the programmatic management of drug-resistant tuberculosis [29].The treatment of MDR-TB requires a long lasting drug exposure, and is also significantly associated with high rates of adverse drug events. Recent meta-analysis report documented a lower treatment success rate among patients treated with a longer MDR-TB regimen compared to those treated with a short course MDR-TB treatment [9].

To our knowledge, this is the first combined analysis to evaluate the effectiveness of the currently used MDR-TB treatment program in Ethiopia. In the present analysis, the pooled estimate of successful treatment was 59.2% (95%CI, 48.1–70.4), while 23.3% (95%CI, 19.7–27.0%) had poor treatment outcome. The treatment success observed in this analysis is comparable with the results of recently published meta-analyses of the outcomes of MDR-TB treatment with conventional drug regimens. Notably, a 54% treatment success rate for conventional MDR-TB treatment regimen was found in a meta-analysis conducted by Ahuja et al. [9]. Similar MDR treatment outcome was estimated in a meta-analysis conducted by Orenstein et al. [30], and pooled analysis from 21 countries [31], showed 62% successful outcome. Thus, it is suggested that the need to modify the duration and the composition of the current MDR-TB treatment regimens. Because of the lengthy therapy, toxicity and fewer efficacies of second-line anti-TB drugs resulting high rates of an unfavorable outcome subsequently lead to the rapid emergence of the extensively drug-resistant TB.

Most recently, an effective standardized treatment regimen lasting less than 12 months has been adopted in countries like Bangladesh, Benin, Burkina-Faso, Burundi, Cameroon, Central African Republic, Côte d’Ivoire, DR Congo, Guinea, Niger, Rwanda, Senegal, Swaziland, and Uzbekistan [10–14]. The regimen composed of the initial phase of 4 months therapy with kanamycin, moxifloxacin, prothionamide, clofazimine, isoniazid, pyrazinamide and ethambutol, and followed by 5 months of treatment with moxifloxacin, clofazimine, pyrazinamide, and ethambutol. Based on the data from recent studies, high treatment success has been achieved in above-mentioned countries. Most importantly, meta-analysis studies revealed that the treatment success rate using shorter MDR/RR-TB treatment regimens was ranged from 83.0–83.7% [6, 7], which higher than previously appreciated treatment outcome. Furthermore, an observational survey of nine African countries also noted promising results of using 12-month regimen [10].

Of note, the latest advancement asserted that the effectiveness of the shorter treatment regimen, in May 2016 WHO moves one step forward to update drug-resistant TB treatment guideline, and underscored the recommendation on the use of the 9–12 month treatment regimen [15]. This encouraged the National Tuberculosis Program (NTP) of Ethiopia need to test and implement a similar 12-month regimen. Though there are several positives with this regimen, its use could be restricted by the fact that patients with fluoroquinolones or other 2nd line drug resistance, extra-pulmonary tuberculosis, pregnancy and with severe clinical problems are known not to be considered for shorter MDR/RR-TB regimen. In this case, the NTPs need to preserve the conventional treatment approaches.

In sub-group analysis, it also estimated that 46.1% (34.2–58.0) cured, 12.8% (5.7–20.0) treatment completed, 14.3% (11.5–17.2) died, 7.5% (3.7–11.3) lost to follow uped, 1.6% (1.1–2.2%) experienced treatment failure and 25.0% (14.6–35.5) of the patients whose treatment outcome was not evaluated. Particularly, the cure rate indicated in our analysis is substantially inferior to the results reported with a shorter MDR-TB regimen [15]. Aside from the drug-resistant nature of the infection, the low cure rate might be due to lack of sufficient MDR-TB care centers, poor patient adherence, the low performance of NTPs at central and regional levels, poor efficacy and prolonged duration of the treatment. According to WHO 2017 report [2], achieving high cure rate one of the key pillars of End TB strategy. The result of this study informs the NTPs to update the current treatment approaches as per recently recommended treatment guideline. However, prior to this prospective cohort analysis of a large number of patients required to support the evidence detailed above. Additionally, the Ethiopian Federal Ministry of Health should design smooth platforms to facilitate surveillance data on culture-based drug susceptibility testing; it may be used to identify a population of eligible patients for Short course MDR/RR-TB treatment.

### Limitations of the study

One of the limitations of this analysis was observational and few studies have been included to measure MDR-TB treatment outcome. Potential predictors of poor treatment outcome Such as HIV infection and history TB treatment was not evaluated because the association was not measured in the original studies. Besides, sub-group analysis of the MDR-TB outcome was not done based on the mode of patient treatment (hospital and/or ambulatory) because lack of clear information in the reports.

## Conclusion

The results suggest that low treatment success rate was estimated among MDR-TB patients whose composition of the treatment was based on a conventionally used treatment regimen. Therefore, our analysis remarks the need to test and implement recently adopted a shorter MDR-TB treatment regimen. Prior to implementation the levels of evidence need to be supported through rigorous institutional and national-wide studies.

## Additional files

Additional file 1: **Table S1.** PRISMA 2009 checklist. (PDF 116 kb)

Additional file 2: **Table S2.** The quality assessment of the included studies into the present meta-analysis (DOCX 14 kb)

## Abbreviations

CI: Confidence interval
HIV: Human immuno-deficiency virus
MDR-TB: Multidrug resistant Tuberculosis
NTPs: National Tuberculosis Control Programs
TB: Tuberculosis
WHO: World Health Organization
