# Supplementary material for: Current treatment of multidrug resistant tuberculosis in Ethiopia: an aggregated and individual patients’ data analysis for outcome and effectiveness of the current regimens
Source: BMC Infect Dis. 2018 Sep 27;18:486. doi: 10.1186/s12879-018-3401-5 (PMC6161354; doi:10.1186/s12879-018-3401-5)
Supplement: Supplementary file 2 — Table S2. The quality assessment of the included studies into the present meta-analysis (DOCX 14 kb) [file 12879_2018_3401_MOESM2_ESM.docx]

Table S2: The quality assessment of the included studies into the present meta-analysis

| **Study name** | **Selection**  **(5 stars)** | **Comparability**  **(2 stars)** | **Outcome**  **(3 stars)** | **Score** |
| --- | --- | --- | --- | --- |
| Baye *et al*, 2018 | *** | * | ** | 6 |
| Girum *et al*, 2017 | **** | * | ** | 7 |
| Alene *et al*, 2017 | **** | * | ** | 7 |
| Mequanint *et al*, 2014 | **** | * | ** | 7 |
| Tolera *et al*, 2018 | *** | * | ** | 6 |
| Meressa *et al*, 2018 | **** | * | ** | 7 |
